# Understanding the Temporal, Regional, Demographic, and Policy Factors Influencing Counties’ Daily Traffic Volume Reductions in Response to COVID-19

**DOI:** 10.1177/03611981211009541

**Published:** 2021-07-22

**Authors:** Mitchell Fisher, Jeffrey J. LaMondia

**Affiliations:** 1 Department of Civil and Environmental Engineering, Auburn University, Auburn, AL

## Abstract

This research aims to understand temporal, regional, demographic, and policy factors that influenced travel reduction within the contiguous United States during the early period of the COVID-19 pandemic. Particularly, this research combines U.S. Census data, infection rates, and state-level mandates to determine their effects on daily, county-level vehicle miles traveled (VMT) estimations from March 1, 2020 to April 21, 2020. Specifically, this work generates metrics of VMT per capita, daily change in VMT, and VMT immediate reaction rates for every county in the U.S.A. and develops regression models to determine how these factors influence VMT rates over time. Results show that state-mandated orders were deployed in a pattern relative to their expected economic impact. Model results showed infection rates may have had a greater influence on forcing state policy adoption, ensuring reduced VMT, rather than the number of cases directly influencing individual travel to a significant degree. Additionally, counties with higher populations or labeled as urban counties saw a greater reduction in VMT across all three models compared with lower population and rural counties. Planners and policy makers in the future can utilize the results of this research to make better informed responses as well as to know the expected results of their actions.

The COVID-19 pandemic has resulted in federal-level mandates and responses, economic turmoil, and disruption of daily operations; all within an unprecedented short timeframe. While the medical aspect of this event cannot be understated, there is still much interest in the pandemic’s effect on the transportation sector, particularly its effects on daily travel numbers in the U.S.A. Not only is this important for understanding potential viral spread, but also for understanding how fast U.S. citizens reacted to new government policies and for future resiliency planning.

Although there has not been a similar recent biological event to compare directly, a rapid change in travel trends was observed and well documented after the September 11, 2001, terrorist attacks—particularly among the tourism and airline industries. In fact, several sources, including Southwest Airlines, have compared the impact of COVID-19 on the airline industry with that of the 9/11 attacks (*1*, *2*). Bonham et al. (*3*) described this phenomenon in general terms where an “external shock” event, such as 9/11 or in this case government quarantine orders, resulted in a rapid downward spike in travel activity. Figure 1 illustrates the idealized recovery process. It should be noted that this acts as a general visualization of the expected trend. Since the COVID-19 pandemic is a more drawn out “shock” event, the recovery slope would not be expected to happen as extremely or suddenly after the initial societal shock.

**Figure 1.**
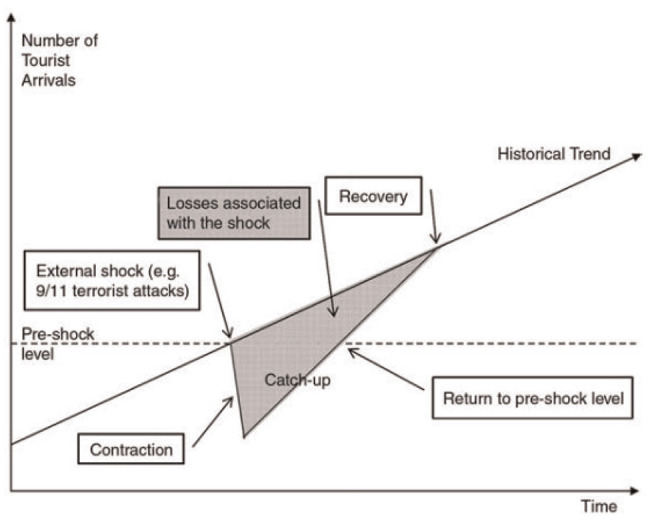
Schematic representation of tourism downturn and recovery (*3*).

The period of recovery to pre-shock levels begins almost immediately until “full recovery”/normality is achieved. In the case of 9/11, international tourism to the U.S.A. saw a sharp decline (*3*) and there was a 20% drop in domestic air travel over the September–December 2001 period (*4*). It has been suggested that pre-9/11 domestic air travel rates never fully recovered, as a result of changes in opportunity costs, benefits, mode shifts, and unrelated economic turmoil (*4* [Bibr bibr5-03611981211009541][Bibr bibr6-03611981211009541][Bibr bibr7-03611981211009541]–*8*), however, the long-term impacts are debated (*9* [Bibr bibr10-03611981211009541][Bibr bibr11-03611981211009541][Bibr bibr12-03611981211009541]–*13*).

Unfortunately, it is not known if the same impact and recovery cycle (well studied in the airline industry) could also apply to daily vehicle travel during the pandemic. This is a critical concern because, while this reduction in travel has generated some benefits (e.g., reduced emissions [*14*] and congestion [*15*]), it has also generated a significant loss of transportation capital associated with local, state, and federal gas excise tax revenue. For example, the federal gas tax loss at the height of COVID-19 vehicle travel reductions was estimated to be $43.5 million per day (*16*). By understanding the factors influencing travel reduction, decision makers could adjust policy accordingly.

Therefore, this research aims to understand temporal, regional, demographic, and policy factors that influenced travel reduction within the contiguous United States during the early period of the COVID-19 pandemic. Particularly, this research combines U.S. Census data, infection rates, and state-level mandates to determine their effects on daily, county-level vehicle miles traveled (VMT) estimations from March 1, 2020 to April 21, 2020. Planners and policy makers in the future can utilize the results of this research to make better informed responses as well as know the expected results of their actions.

The paper is organized as follows: first, the methodology used to determine VMT as well as the actual measured variables is presented, along with general descriptive statistics of the other input data. Next, observed trends in travel reduction are discussed and explored. This is followed by the methodology and results of the three VMT estimation models. Last, conclusions on general findings and suggestions for future research are submitted.

## County VMT and Influencing Factor Data

VMT is the total number of miles every vehicle in a geographic region completes in a given timeframe (*17*). Transportation researchers and planners rely on VMT to indicate accurately a region’s travel demand as well as temporal travel behavior. While VMT can be isolated to individual roadways, it is usually presented as a single value for an entire geographic region; for this research, the base geographic scale is the county (or county-equivalent) level. To characterize changes in the VMT rate, sociodemographic county data, COVID-19 case rates, and state-level pandemic response actions were collected and related to the relevant VMT data.

This section is structured as follows: first, the methodology used for the VMT data as well as its source methodology is presented. Next, additional county characteristics are discussed as well as a timeline of federal and state pandemic response policies. Finally, COVID-19 case rates are briefly covered.

### Characterizing VMT with Three Metrics

County VMT data was acquired from StreetLight Data in their effort to provide daily VMT estimations, at no cost, to researchers during the COVID-19 pandemic. Specifically, this study analyzes daily VMT during the preliminary shock period: March to April 2020. While peak hour VMT would also be interesting to consider, daily VMT was the highest resolution available at this time. StreetLight generates VMT data at many spatial scales for planners across the country based on GPS data collected from cell phone navigation-GPS data and location-based services data. These data sources (INRIX and Cuebiq, respectively) provide StreetLight with millions of de-identified GPS points following about 23% of all individuals traveling on roadways across the U.S.A. each day. StreetLight then follows chains of GPS points to identify specific trips within the data, including origins, destinations, routes, and travel times. These sampled trip volumes are then scaled to mimic the full traffic patterns using U.S. Census and American Community Survey benchmarks. For this dataset, StreetLight validated estimated VMT with observed VMT for the month of February (with an R^2^ of 0.98) (*18*). A noted caveat of this method is that the calculated VMT will only reflect VMT of individuals residing in the county and not those just passing through the county. Additionally, counties with a sample size of less than 500 daily trips or a VMT less than 5,000 were removed from the final dataset.

To best utilize this data source, the authors calculated three VMT metrics to further compare and describe the trends among counties: VMT per capita, daily change in VMT, and VMT immediate reaction rate.

#### VMT per Capita

Since VMT is heavily correlated with population (basic linear regression of county population and average January 2020 VMT produces an R^2^ of 0.862), a better way to compare VMT among different geographic areas is to divide VMT by the population, known as VMT per capita. For this analysis, daily county VMT was divided by the population of the county or county-equivalent. Units for this metric are described as “miles per person.”

#### Daily Change in VMT

The daily change in VMT was calculated to capture the percent change in VMT relative to March 1, 2020, using the standard percent change formula.

(1) $$\Delta_{\mathrm{VMT}}=\frac{\mathrm{VM}T_{i}-\mathrm{VM}T_{1\mathrm{March}}}{\mathrm{VM}T_{1\mathrm{March}}}\times 100$$

where *Δ_VMT_* is the daily change in VMT (%); *VMT_i_* is the VMT of the target day, *i*; and *VMT_1 March_* is the VMT on March 1, 2020, for that county. This metric was calculated daily on the county-level and could be either positive (an increase in VMT) or negative (a decrease in VMT) based on the county or day.

#### VMT Immediate Reaction Rate

In an effort to capture the response of the public better, a decision was made to focus on VMT from March 13 to March 27. These dates were chosen based on relevance to major events (March 13 was two days after COVID-19 was declared a pandemic by the World Health Organization) and VMT trend analysis (both dates represent major milestones: the peak average VMT [March 13] and the “new normal” VMT [March 27]). To quantify this reaction rate, the rate of change, or slope, of county VMT over time was calculated for each county. Most counties displayed linear reduction rates between these dates (as opposed to the normal weekly fluctuation trends) making this method an ideal candidate for simplistically quantifying the concept of a reaction rate. In other terms, the VMT immediate reaction rate measures how quickly a county’s travel behavior changed from pre-pandemic status to the “new normal.”

**Figure 2.**
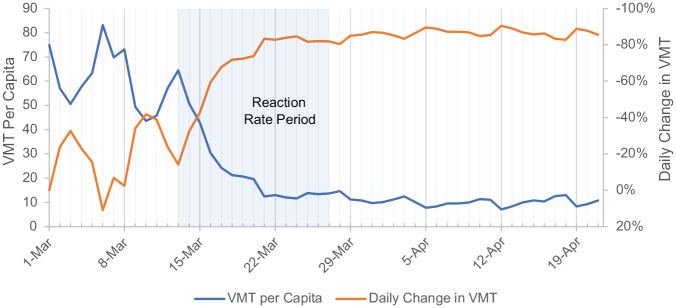
Example of vehicle miles traveled (VMT) trends: Fulton County, Georgia.

An example of the three VMT metrics for Fulton County, Georgia, where the city of Atlanta is located, can be seen in Figure 2. Here, daily VMT noise as well as major milestones are clearly presented. For Fulton County, in particular, there was a noted spike in VMT on Friday, March 6, before overall VMT began to trend down over the next 18 days. At this point, the “new normal” VMT weekly trends begin with the weekly peaks on Fridays still present and a reduction of up to 90% in VMT from March 1 is observed. The immediate reaction rate analysis period is highlighted within the blue box. Note the steep decline in VMT per capita over the time period. Similar trends were seen in the majority of counties, especially urban counties.

### Supporting County Characteristics

To characterize each county beyond its daily VMT values, relevant census information and accessibility metrics were compiled from Census Transportation Planning Products, American Community Survey 5-Year Estimation data (*19*). Each county was characterized by census division, urban/rural population majority, median household income, population, and percent previously working from home. To help capture more intangible county concepts, three long-distance accessibility metrics (economic, leisure, and air) were also calculated to describe the regional importance, mobility, and connectivity of each county. These metrics are a conglomeration of different access-centric, or “opportunity,” measures in long-distance travel, attractions, and economic viability, validated from previous work (*20*). These metrics were selected over others (e.g., transport subsidies, transportation mode availability) because they used gravity model accessibility equations to combine (a) ease of access to surrounding metropolitan areas and (b) the relative attractiveness of these metropolitan areas into comprehensive measures. Particularly:

- *Economics*: Measurement of the “intensity” of specialized industries in a metropolitan statistical area (MSA). Specialization, also known as a location quotient, is determined by the proportion of area employment in a specific industry to the national proportion. Areas with higher scores of this variable are theorized to attract a higher long-distance travel demand for work.
- *Leisure*: Measurement of the “intensity” of leisure specialization (arts, recreation, and entertainment industries) within an MSA. Determined by the same methodology used in the economic calculation. Areas with a higher leisure score, such as Las Vegas, would have heightened tourist activity.
- *Air*: Measurement of annual enplanements. Higher passenger activity suggests greater long-distance attractiveness.

These values range from 0 to 100, with counties scoring zero having the least amount of long-distance accessibility/opportunity and counties scoring 100 having the greatest.

As noted previously, counties with either fewer than 500 daily trips or a VMT of 5,000 were dropped from the dataset. In addition to these dropped counties, only the contiguous United States was considered for this research. As such, Alaska and Hawaii counties/county-equivalents were also removed from the final dataset. Table 1 presents means and standard deviations of the counties in the study for each census division.

**Table 1.** Summary Statistics of County Characteristics by U.S. Census Division

	Statistic	New England	Middle Atlantic	East North Central	West North Central	South Atlantic	East South Central	West South Central	Mountain	Pacific^a^
% counties represented		94.0	97.3	96.8	77.7	95.4	97.5	92.1	69.8	89.4
# counties in study		63	146	423	480	561	355	433	196	122
% urban counties in study		47.6	59.6	43.3	37.5	41.4	26.5	42.3	64.8	80.3
Median income ($K)	Mean	$59.49	$57.13	$50.06	$50.45	$46.61	$39.48	$44.99	$51.06	$53.98
	SD	$12.707	$14.460	$9.111	$9.352	$14.778	$9.595	$10.374	$13.445	$13.454
% previously working from home	Mean	5.63	4.11	3.96	5.37	3.86	2.88	3.31	6.04	6.15
	SD	1.632	1.270	1.393	2.519	2.207	1.406	1.624	2.690	2.117
Population (1,000)	Mean	232.19	283.38	110.18	42.85	111.13	52.80	88.59	116.63	407.11
	SD	307.621	418.670	310.558	103.376	227.764	95.838	299.928	357.112	1055.63
Long-distance access score										
Economics	Mean	42.40	67.44	67.14	51.78	65.88	68.33	53.39	23.57	19.32
	SD	19.970	17.759	21.907	21.576	18.498	18.085	16.605	18.566	18.647
Leisure	Mean	36.84	50.99	41.21	30.30	45.55	39.98	26.15	13.44	17.91
	SD	15.225	14.819	14.967	14.108	13.286	11.910	12.464	11.042	9.919
Air travel	Mean	7.70	9.06	3.36	2.41	5.25	2.36	2.78	2.06	5.55
	SD	11.017	18.223	5.820	4.435	8.230	4.025	4.945	5.152	8.942

*Note*: SD = standard deviation.

a Only contiguous states (Alaska and Hawaii excluded).

County representation on the census division level saw lows of 69.8% for the Mountain Division and 77.7% for the West North Central Division. This was mainly because of the rural nature of Montana, North Dakota, and South Dakota; all of which had less than 45% representation. The average state representation percentage was roughly 89%.

#### Federal and State Policies

Over the course of the pandemic, governmental bodies on all levels have been implementing public measures to help slow the spread of the virus. While numerous cities and counties enacted their own mandates, the decision was made to see how state and federal policies affected the public reaction because (a) the sheer variety of city/county-level actions could cause minimum sample size issues, and (b) there was no efficient way to catalog and research these policies for data deployment. Utilizing the Institute for Health Metrics and Evaluation’s (IHME) COVID-19 models, a timeline of social distancing measures on the state level was gathered and added to the VMT data (*21*). The IHME classified government-mandated social distancing measures into six distinct types based on the New Zealand Government’s Alert System Level 4 (*22*):

- *Severe Travel Restrictions*: Borders are closed to all non-essential traffic except returning residents. Public transit is closed, and automobile travel is limited to accessing essentials only.
- *Stay at Home Order*: All individuals must shelter in place except for accessing essential services. Physical contact is limited to only members of the same household. There are fines or prosecution orders in place for non-compliance.
- *Initial Business Closures*: Time when any business type was closed due to containment measures, particularly bars or restaurants.
- *Mass Gathering Restrictions*: Any mandatory restrictions on public or private gatherings such as limiting party sizes to 10 individuals.
- *Education Facilities Closed*: All education levels have implemented distance learning measures and closed in-person teaching.
- *Non-Essential Business Closures*: All businesses deemed “non-essential” are closed from public use such as bars, restaurants, retail stores, and public spaces. Any location where social distancing measures are impractical are also closed. There is an enforceable consequence for non-compliance.

In gathering this information, it is important to note that all actions (excluding travel restrictions) were enacted on the state level. On the federal level, only international travel restrictions were implemented, with all other measures being left to state governments to enact. Table 2 presents the timelines for different policy implementations across the country.

**Table 2.** Federal and State COVID-19 Response Policy Implementation Dates

	State	International travel restrictions	Stay at home order	Initial business closure	Mass gathering restriction	Education facilities closed	Non-essential business closure
Federal		2/2	na	na	na	na	na
New England	CT	na	na	3/16	3/12	3/17	3/23
	MA	na	**4/2**	3/18	3/18	3/16	3/25
	ME	na	na	3/17	3/13	3/17	3/24
	NH	na	3/27	3/16	3/16	3/16	3/28
	RI	na	3/28	3/17	3/17	3/16	na
	VT	na	3/24	3/17	3/13	3/18	3/25
Middle Atlantic	NJ	na	3/21	3/16	3/16	3/18	3/21
	NY	na;	3/22	3/16	3/12	3/18	3/22
	PA	na	**4/1**	3/18	**4/1**	3/17	3/23
East North Central	IL	na	3/21	3/16	3/13	3/17	3/21
	IN	na	3/25	3/16	3/12	3/19	3/24
	MI	na	3/24	3/16	3/13	3/16	3/23
	OH	na	3/23	3/15	3/12	3/16	3/23
	WI	na	3/25	3/17	3/17	3/18	3/25
West North Central	IA	na	na	3/17	3/17	**4/3**	na
	KS	na	3/30	na	3/17	3/17	na
	MN	na	3/27	3/17	3/24	3/18	na
	MO	na	**4/6**	3/23	3/23	3/23	na
	ND	na	na	3/20	na	3/16	na
	NE	na	na	3/19	3/16	**4/2**	na
	SD	na	na	na	**4/6**	3/16	na
	State	International travel restrictions	Stay at home order	Initial business closure	Mass gathering restriction	Education facilities closed	Non-essential business closure
South Atlantic	DC	na	3/30	3/16	3/13	3/16	3/25
	DE	na	3/24	3/16	3/16	3/16	3/24
	FL	na	**4/3**	3/17	**4/3**	3/17	na
	GA	na	**4/3**	3/24	3/24	3/18	na
	MD	na	3/30	3/16	3/16	3/16	3/23
	NC	na	3/30	3/17	3/14	3/14	3/30
	SC	na	**4/7**	3/18	3/18	3/16	na
	VA	na	3/30	3/17	3/15	3/16	na
	WV	na	3/25	3/18	3/24	3/14	3/24
East South Central	AL	na	**4/3**	3/19	3/19	3/19	3/28
	KY	na	na	3/16	3/19	3/20	3/26
	MS	na	**4/3**	3/24	3/24	3/19	**4/3**
	TN	na	**4/2**	3/23	3/23	3/20	**4/1**
West South Central	AR	na	na	3/19	3/27	3/17	na
	LA	na	3/23	3/17	3/13	3/16	3/22
	OK	na	na	**4/1**	3/24	3/17	**4/1**
	TX	na	**4/2**	3/21	3/21	3/19	na
Mountain	AZ	na	3/30	na	3/30	3/16	na
	CO	na	3/26	3/17	3/19	3/23	3/26
	ID	na	3/25	3/25	3/25	3/23	3/25
	MT	na	3/26	3/20	3/24	3/15	3/26
	NM	na	na	3/16	3/12	3/13	3/24
	NV	na	3/31	3/18	3/24	3/16	3/20
	UT	na	na	3/19	3/19	3/16	na
	WY	na	na	3/19	3/20	3/19	na
Pacific	AK^a^	na	3/28	3/17	3/24	3/16	3/28
	CA	na	3/19	3/19	3/11	3/19	3/19
	HI^a^	na	3/25	3/17	3/17	3/19	3/25
	OR	na	3/23	3/17	3/12	3/16	na
	WA	na	3/23	3/16	3/11	3/13	3/25

*Note*: April dates in bold. na = not applicable (this policy was not implemented in that state).

a Not included in final analysis.

**Figure 3.**
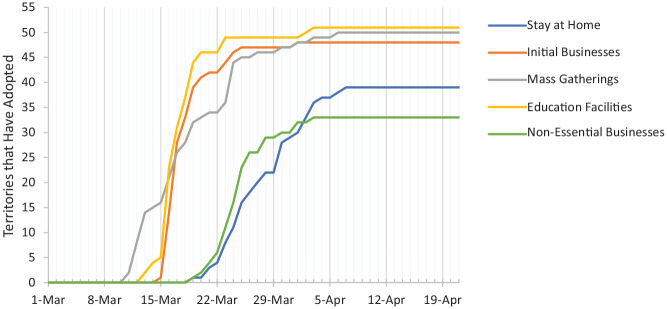
Timeline of orders (states plus District of Columbia).

By April, all states had implemented at least one of the five social distancing measures. What is interesting is how states in the same census division reacted similarly. For example, none of the states in the West North Central division closed non-essential businesses, and states in most divisions enacted stay at home orders around the same time. Figure 3 visually illustrates the general timeline of each order.

Orders followed two distinct groupings, both heavily dependent on their economic impact potential. The first grouping consists of the initial business closures, education closures, and mass gathering restrictions. These policies are the least intrusive and economically harmful of the options and were implemented first. By April, almost all states and territories had enacted these policies. The spike around March 16 is possibly explained by the Trump Administration recommending school closures and limiting mass gatherings. For reference, March 11 was the day COVID-19 was declared a pandemic by the World Health Organization. The second grouping of orders were the stay at home and non-essential business closures. As these orders were considered the most severe with regard to economic impact and personal freedom, their late adoption could be expected. In fact, these orders were never fully adopted by all states and territories but were adopted by the majority.

#### COVID-19 Infection Rates

Finally, daily infection data at county and state level were collected from USAFacts (*23*). Their methodology aggregated infection data from Center for Disease Control and Prevention and state/local-level health agencies. This data was validated by USAFacts by contacting state and local agencies directly. Each county was assigned a daily cumulative total with indeterminate cases (cases where a lack of information prevents county assignment) counted among the state-level cumulative total. For this analysis, each county was given two daily infection rates: county-level and state-level. By including both rates in the analysis, it would be possible to see if the county population reacted to more localized numbers or regional rates.

## VMT Traffic Reduction Trends

While statistical modeling allows for a more in-depth understanding of the causes of VMT change, visually representing VMT trends can still offer substantial information and relationships that are difficult or not necessarily possible to see via modeling. A variety of trends were considered, but it was decided to focus on four main facets: daily VMT versus the total number of COVID-19 cases, VMT trends in relation to policy enactment, daily VMT rates for counties that had and had not enacted social distancing measures, and VMT reaction rates by county. Unless otherwise noted, VMT was represented as the median value for the specified grouping (census division or census region level). Median values tend to represent data that has known extremes better than an average value. Since county VMT levels varied wildly, this choice better fits the data.

**Figure 4.**
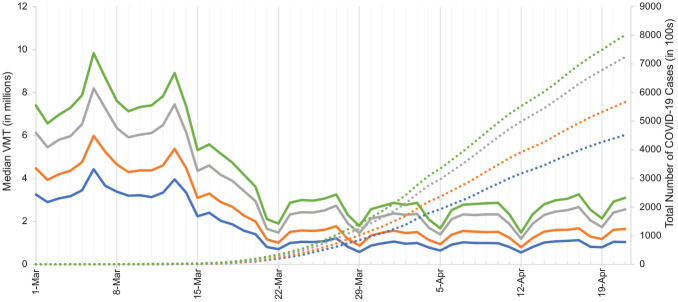
Median daily vehicle miles traveled (VMT) and number of infections by census region. Figure 4 Legend 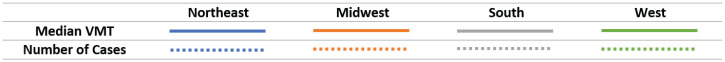

Figure 4 compares median VMTs with total number of COVID-19 cases by census region. Interestingly enough, VMT and the number of cases seemed to have very little overlap with one another with regard to their general trends. The number of cases began to increase exponentially around March 17, and while there is a noticeable decrease in VMT around that time, the severe VMT reduction period was still earlier, beginning March 13 (more in line with policy announcements).

**Figure 5.**
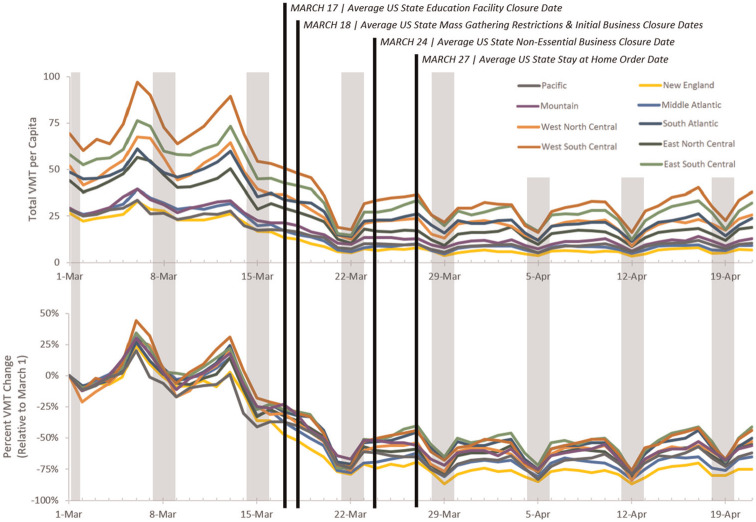
Trends in vehicle miles traveled (VMT) and policies by census division (weekends highlighted in gray).

Further delving into the impact policy had on VMT rates, Figure 5 presents the VMT per capita and percent change in VMT on the census division level with the average date of each policy enactment. Weekends are labeled in blue. VMT per capita showed some differences among the divisions with two distinct groupings present. Presenting VMT as a function of percent change from March 1 (Figure 5*b*) created a tighter grouping, suggesting divisions did react alike when controlling for the lack of data from before March.

**Figure 6.**
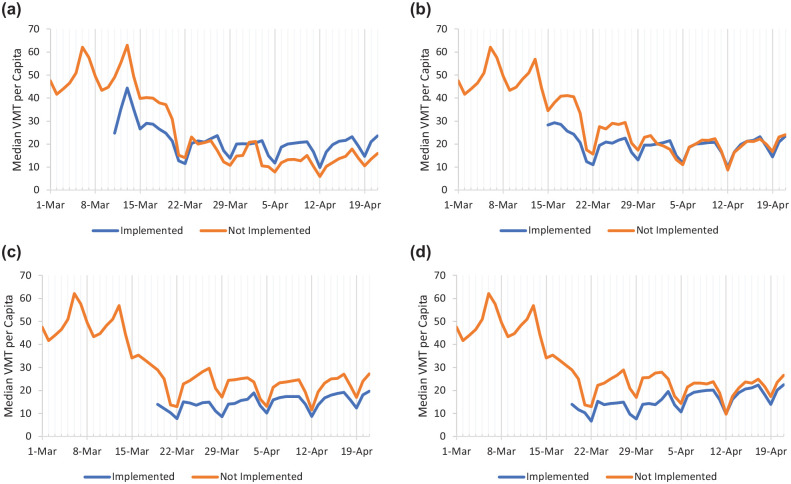
Median vehicle miles traveled (VMT) per capita in reaction to state mandates: (*a*) mass gatherings limited, (*b*) initial business closures, (*c*) non-essentials closed, (*d*) stay at home.

Similar to the early reaction seen in Figure 4, VMT rates began dropping before policies became widely adopted. Recalling Figure 3 confirms this: as of March 13, only two states had closed education facilities and 14 had implemented mass gathering restrictions. This would suggest other external forces had a role in shaping reaction; be it local/city-level orders, self-closures of education facilities or businesses, or the news cycle. What could be evident is that mandatory stay at home and non-essential business closures may have had very little effect on overall VMT reduction. To explore this claim further, counties were divided into two groups: those that had implemented a policy and those that had not. VMTs were then adjusted for population and the median values graphed. Figure 6 presents each of the four policies where there was a lack universal adoption across states. For all four policies, states with implementation saw a significant initial decrease in VMT compared with counties that had yet to implement the policy. However, this leads to a chicken-and-egg situation: were policies enacted proactively or reactively? Regardless, as time went on, the effectiveness of each policy on reducing travel became apparent. Each policy did reduce VMT per capita compared with non-implementing counties, but as the pandemic wore on, mass gathering policies severely lost their effectiveness as well as the stay at home and initial business closure policies. What did remain effective was the closure of non-essential businesses. While economically taxing, completely removing the option of physically visiting these businesses appeared to sizably reduce median VMT per capita. Surprisingly, this effect was not as prominent with the initial business closures; however, as these businesses were mostly bars and restaurants, this could be the result of adoption of “to-go” and delivery business models.

**Figure 7.**
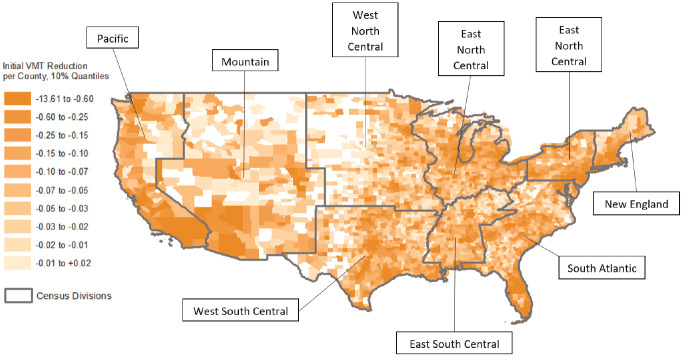
Vehicle miles traveled (VMT) reaction rates by county (census division outlined in gray).

Figure 7 visualizes the VMT reaction rate of each county on a gradient scale; highlighting the major influence of urban locale on immediate VMT reduction. Counties shown in white are those where no data, or incomplete data, were available. As reaction rate was calculated as the slope in VMT change between March 13 and March 27, the influence of population would be controlled. However, while urban counties generally had greater reaction rates, some less urbanized counties exhibited reaction rates just as extreme. This visualizes the idea that VMT reaction to the pandemic was influenced by a myriad of factors. For example, states where policies were enacted earlier—such as California, New York, Oregon, and Washington—had more uniform reaction rates regardless of county urbanization.

## Factors Influencing VMT Traffic Reduction Trends

For each of the three VMT dependent variables (VMT per capita, VMT percent change, VMT reaction rate), a mixed multivariate linear model was applied to determine what factors best influenced each county’s VMT values. The mixed multivariate linear model functions similarly to a standard linear regression model but takes into consideration the variations of repeated observations. As the VMT data was a time-series plot of each county and each county reacted differently, declaring the counties as the “subjects” of the model would provide a more accurate representation of what factors influenced VMT values. To simplify the models further, as well as control for the observed weekly VMT fluctuation patterns, county VMT data for the VMT per capita and VMT percent change models was reduced to two datasets: Friday-only observations and Sunday-only observations. Preliminary testing concluded the Friday-only models outperformed the Sunday-only models significantly.

Each model was run with all the sociodemographic variables shown in Table 1, daily totals of COVID-19 cases, and a running total of “days since” a particular government action was enacted. Since the VMT reaction rate model was run with a single observation for each county, the number of COVID-19 cases were represented as the March 27 totals and each government action was observed as a binary variable: 0 if action was not taken by March 27, and 1 if action was implemented by March 27. Goodness of fit was measured using −2 log likelihood and Akaike Information Criteria (AIC) values. Table 3 presents the results for each model.

**Table 3.** Estimation Coefficient Results

	Friday VMT per capita	Percent Friday VMT change	Immediate VMT reaction rate
Model performance			
−2 Log likelihood	195296.775	196453.655	1838.508
AIC	195336.775	196493.655	1874.508
Intercept	149.883^***^	5.979	−2.057^***^
Number of COVID-19 cases			
State (100,000s)	−4.959^***^	−18.200^***^	−0.348^**^
County (100,000s)	na	na	25.400^***^
County characteristics			
Urban (relative to rural)	na	−8.790^***^	−0.156^***^
Median income ($K)	0.087^***^	−0.366^***^	−0.002^***^
Population (100,000s)	−0.609^***^	−0.491^***^	−0.164^***^
% previously working from home	−61.611^***^	−74.717^***^	−1.298^***^
Economic LD accessibility	0.122^***^	na	na
Leisure LD accessibility	−0.188^***^	0.090^***^	na
Air travel LD accessibility	0.109^***^	−0.088^**^	na
Census division			
New England	na	−6.161^***^	−0.195^***^
Middle Atlantic	na	na	−0.183^***^
East North Central	9.939^***^	−3.202^***^	−0.372^***^
West North Central	17.520^***^	−3.668^***^	−0.280^***^
South Atlantic	23.715^***^	5.319^***^	−0.327^***^
East South Central	26.913^***^	na	−0.419^***^
West South Central	39.502^***^	6.207^***^	−0.428^***^
Mountain	6.175^***^	6.262^***^	−0.236^***^
Pacific (base)	na	na	na
Days since state order issued (squared)…			
Stay at home order	0.037^***^	0.0625^***^	na
Initial business closure	−0.010^***^	−0.011^***^	na
Mass gathering restriction	−0.010^***^	−0.027^***^	na
Education facilities closed	−0.025^***^	−0.0416^***^	na
Non-essential business closure	0.007^**^	0.008^***^	na
State order issued during immediate timeframe			
Stay at home order	na	na	na
Initial business closure	na	na	na
Mass gathering restriction	na	na	0.110^***^
Education facilities closed	na	na	na
Non-essential business closure	na	na	0.096^***^

*Note*: AIC = Akaike Information Criteria; LD = long distance; na = not applicable; VMT = vehicle miles traveled.

* 90% Confidence Level.

** 95% Confidence Level.

*** 99% Confidence Level.

Across all three models, the number of COVID-19 cases in the county or state, while statistically significant, had very little actual effect on their respective dependent variables. Furthermore, with the exception of the reaction rate model, state COVID-19 totals were the statistically significant influencer. This could suggest that the state totals had a larger effect on forcing state policy adoption, ensuring decreases in VMT, rather than the number of cases directly influencing individual travel to a significant degree.

County demographics show that urban county, high population, and the percent previously working from home had universal negative effects on VMT volumes. The effects of percentage of population previously working from home are self-explanatory: these individuals had little to no daily commute, limiting their contributions to county VMT totals. Urban designation and population, while directly correlated, still provided two levels of effect for influence on VMT. Higher population areas saw a greater reduction in VMT for all three models, when all other variables were held constant. This was also true for urban counties. As population density has been shown to increase the risk of disease spread (*24*, *25*), higher population areas would possibly be more motivated to reduce unnecessary travel and exposure compared with low density rural counties. County median income results possibly reflected normal travel behavior. While the percent change and reaction rate models showed greater reductions in VMT volumes with an increase in median income, the VMT per capita model showed an increase in VMT volumes. As income had been proven to directly influence VMT per capita (26), higher median income counties would have a higher base VMT per capita value than lower income counties.

Long-distance accessibility scoring showed no statistical influence on VMT reaction rates, but mixed results for the VMT per capita and percent change models. The VMT per capita model was affected positively by the economic and air scoring while being affected negatively for leisure scoring. This would suggest that industry specialization, possibly those deemed “essential industries,” saw little change in daily operations, while counties that relied more on leisure industry specialization did see a drop in activity. However, the percent change model saw the opposite effects with regard to leisure and air accessibility scoring. As VMT per capita was defined as daily VMT divided by county population, this could suggest leisure-related long-distance travel into the county saw a decline (VMT per capita), but localized travel was still prevalent. Air accessibility score effects present an increase in VMT per capita but a decline in VMT percent change. Once again, this could be because areas with higher passenger enplanements had a higher base level for VMT per capita, but still experienced a decline in daily VMT based on percent change.

The results of the reaction rate model provided the best representation of geographic VMT response. Relative to the Pacific division, the West South Central and East South Central divisions had the steepest decline in VMT during the reaction period. Comparatively, the New England and Middle Atlantic divisions had milder declines. However, this could be the result of pre-reaction VMT levels being generally greater for both the West South Central and East South Central divisions. While all divisions did show rapid reaction rates during the same timeframe, data limitations did not provide more information about traffic volumes in the period before the COVID outbreak.

VMT response to different state-issued mandates provided some of the most interesting results. The VMT per capita and percent change (time-series) models suggested that the closure of education facilities had the greatest impact on reducing VMT volumes among all other state orders. This was followed by restricting mass gatherings and initial business closures. However, both stay at home and non-essential business closures saw a slight *increase* in VMT volumes when compared directly with all other state orders. As these orders were implemented generally later into the pandemic, past the initial “shock,” their impact might have been affected by the lack of available VMT capital. This same logic proves true for why education closures had the greatest negative effect: as the closure of education facilities was, on average, the first state-issued order (referring to Figure 5), the available VMT capital before reaching the “new normal” was greater, which resulted in a larger negative effect on VMT. The results of the reaction rate model produced notably different effects. Counties with both mass gathering restrictions and non-essential business closure saw a slight increase (less steep slope) to the VMT reaction rate. This does not necessarily conclude that these policies had an adverse effect on VMT reduction, but may simply reflect the already lower daily VMT volumes before adoption of the orders. This is supported in Figure 6, where the counties that have implemented the order started from lower VMT per capita values. Overall, it is suggested that every government order had some impact on VMT levels to some degree, but it cannot be concluded if any order had a solely adverse effect on travel.

## Conclusions

The 2020 COVID-19 pandemic has affected daily life on a scale rarely seen before, but with advances in technology researchers have the ability to actively monitor and study the effects of the pandemic on the medical, economic, and societal front as never before. This study aimed to measure the impact of COVID-19 on daily travel trends. In particular, daily county VMT data from March 1 to April 21 was nationally collected and analyzed for the effects of government actions, infection rates, and sociodemographics on travel response. It is hoped that this research will provide insights for future planners on policy effectiveness as well as expected daily travel response to sudden-shock level events.

The results show state-mandated orders were deployed in a pattern relative to their expected economic impact. Low-impact policies such as limiting mass gatherings and restricting certain businesses (bars and restaurants) were enacted first, before policies having greater impact, like non-essential business closures and stay at home orders. Additionally, only the closure of education facilities was universally adopted by all states and territories. Analysis and comparison of VMT between states that implemented and did not implement orders showed that each policy did reduce VMT per capita compared with non-implementing counties, but as the pandemic wore on, mass gathering policies severely lost their effectiveness, as did the stay at home and initial business closure policies. What did remain effective was the closure of non-essential businesses. While economically taxing, completely removing the option of physically visiting these businesses appeared to sizably reduce median VMT per capita.

The model results show that infection rates may have had a greater influence on forcing state policy adoption, ensuring drops in VMT, rather than the number of cases directly influencing individual travel to a significant degree. Additionally, counties with higher populations or labeled as urban counties saw a greater reduction in VMT across all three models compared with lower population and rural counties. State-mandated orders had an unclear effect on VMT values in the context of the models. While policies of stay at home and non-essential business closures saw a slight increase in VMT per capita, their late adoption among the majority of states resulted in a lack of available “VMT capital” which lessened their apparent effectiveness. This same logic proves true for why education closures had the greatest negative effect: as the closure of education facilities on average was the first state-issued order, the available VMT capital before reaching the “new normal” was greater, which resulted in a larger negative effect on VMT.

While the results from this research do highlight the immediate reactions to the COVID-19 pandemic, this research was conducted during the pandemic, limiting the possible scope of measured economic, societal, and psychological impacts. Future work should consider the long-term impacts of reduced VMT, gas revenues, and associated business activity on local, state, and regional economies.

Additionally, it was noted that the unavailability of data for preceding months prevented a direct comparison with normal VMT numbers. As such, it is suggested that future research focus on a three-pronged approach: analysis of March–April 2019 VMT data, March–April 2020 VMT data, and a future March–April VMT timeframe to fully understand the effects COVID-19 has had on normal operations as well as VMT recovery. Another suggestion would be further consideration of how local-level (city, MSA, county) policies; such as those in the Northeast census region and Pacific census division, compared with regional, state, and national policies.
